# High prevalence of shoulder girdle muscles with myofascial trigger points in patients with shoulder pain

**DOI:** 10.1186/1471-2474-12-139

**Published:** 2011-06-28

**Authors:** Carel Bron, Jan Dommerholt, Boudewijn Stegenga, Michel Wensing, Rob AB Oostendorp

**Affiliations:** 1 Scientific Institute for Quality of Healthcare, Radboud University Nijmegen Medical Centre, Geert Grooteplein 21, 6525 EX Nijmegen, The Netherlands; 2 Practice for Physical Therapy for Neck, Shoulder and Upper Extremity Disorders, Groningen, Paulus Potterstraat 46, 9718 TK Groningen, The Netherlands; 3 Bethesda Physiocare Inc., 7830 Old Georgetown Road, Suite C-15, Bethesda, MD 20814-2440, USA; 4 Department of Oral and Maxillofacial Surgery, University Medical Centre Groningen, Hanzeplein 1, 9713 GZ Groningen, The Netherlands

**Keywords:** myofascial, pain, trigger points, prevalence, shoulder

## Abstract

**Background:**

Shoulder pain is reported to be highly prevalent and tends to be recurrent or persistent despite medical treatment. The pathophysiological mechanisms of shoulder pain are poorly understood. Furthermore, there is little evidence supporting the effectiveness of current treatment protocols. Although myofascial trigger points (MTrPs) are rarely mentioned in relation to shoulder pain, they may present an alternative underlying mechanism, which would provide new treatment targets through MTrP inactivation. While previous research has demonstrated that trained physiotherapists can reliably identify MTrPs in patients with shoulder pain, the percentage of patients who actually have MTrPs remains unclear. The aim of this observational study was to assess the prevalence of muscles with MTrPs and the association between MTrPs and the severity of pain and functioning in patients with chronic non-traumatic unilateral shoulder pain.

**Methods:**

An observational study was conducted. Subjects were recruited from patients participating in a controlled trial studying the effectiveness of physical therapy on patients with unilateral non-traumatic shoulder pain. Sociodemographic and patient-reported symptom scores, including the Disabilities of the Arm, Shoulder, and Hand (DASH) Questionnaire, and Visual Analogue Scales for Pain were compared with other studies. To test for differences in age, gender distribution, and education level between the current study population and the populations from Dutch shoulder studies, the one sample T-test was used. One observer examined all subjects (n = 72) for the presence of MTrPs. Frequency distributions, means, medians, standard deviations, and 95% confidence intervals were calculated for descriptive purposes. The Spearman's rank-order correlation (ρ) was used to test for association between variables.

**Results:**

MTrPs were identified in all subjects. The median number of muscles with MTrPs per subject was 6 (active MTrPs) and 4 (latent MTrPs). Active MTrPs were most prevalent in the infraspinatus (77%) and the upper trapezius muscles (58%), whereas latent MTrPs were most prevalent in the teres major (49%) and anterior deltoid muscles (38%). The number of muscles with active MTrPs was only moderately correlated with the DASH score.

**Conclusion:**

The prevalence of muscles containing active and latent MTrPs in a sample of patients with chronic non-traumatic shoulder pain was high.

## Background

Shoulder pain, which is often persistent or recurrent, is one of the major reasons patients consult with primary healthcare providers [1-6]. However, the pathophysiological mechanisms underlying shoulder pain are poorly understood. Although subacromial impingement is often suggested to be a potential source of shoulder pain [7,8], solid evidence is lacking. In fact, calcifications, acromion spurs, subacromial fluid, or signs of tendon degeneration are equally prevalent in healthy subjects and in patients with shoulder pain [9-12]. Furthermore, physical examination tests of subacromial impingement are not reliable [13-15], and the results of imaging diagnostics do not correlate well with pain [9,10,16,17]. In addition, interventions targeting subacromial problems are, at most, only moderately effective at treating shoulder complaints [18-24].

Myofascial trigger points (MTrPs) may offer an alternative explanation for the pathophysiological mechanisms underlying shoulder pain. In recent years, our understanding of the etiology, pathophysiology, and management of MTrPs has increased [25-30]. MTrPs are local points, that are highly sensitive to pressure, the application of which causes characteristic referred sensations, including pain, muscle dysfunction [26], and sympathetic hyperactivity [31-33].

MTrPs are classified into active and latent myofascial trigger points. Active MTrPs are characterized by the presence of clinical pain and constant tenderness. Specifically, active MTrPs prevent full lengthening and weakening of the muscle. Diagnostically, active MTrPs refer patient-recognized pain upon compression and mediate a local twitch response in muscle fibers when stimulated. When compressed within the patients' level of pain tolerance, active MTrPs produce referred motor phenomena and often sympathetic hyperactivity, (generally in the pain reference zone), and cause tenderness in the pain reference zone. In contrast, latent MTrPs are clinically quiescent, and are only painful when palpated. With the exception of spontaneous pain, a latent MTrP can present with all the clinical characteristics of active MTrPs. In addition, latent MTrPs are within a taut band that increases muscle tension and restricts patients' range of motion [26]. Although the exact pathophysiology of MTrPs is not yet fully understood, abnormal electrical activity, called endplate noise, has been associated with both latent and active MTrPs, and several pain-inducing and pro-inflammatory substances have been found at active MTrP in humans [27,34].

**Figure 1:**
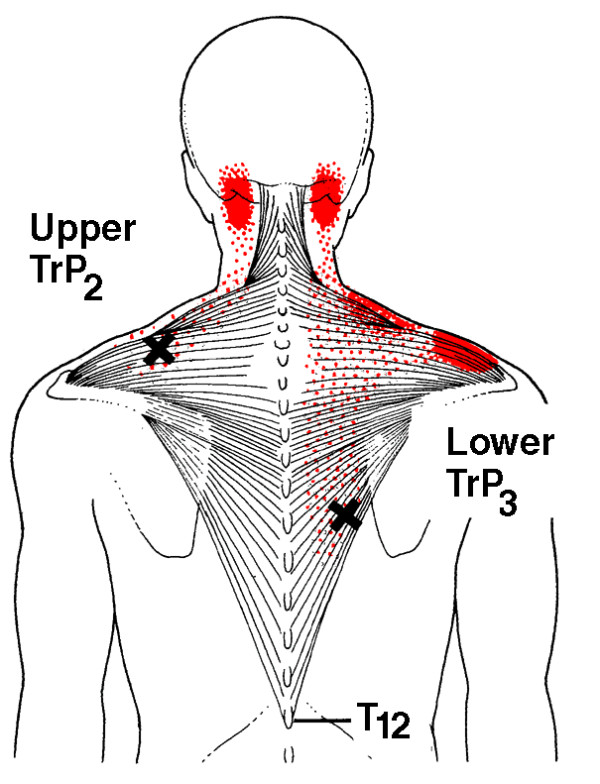
**Referred pain patterns (red) from the upper and lower trapezius muscle MTrPs (Xs), according to Simons et al**. Illustrations courtesy of LifeART/MEDICLIP, Manual Medicine 1, Version 1.0a, Lippincott, Williams & Wilkins, 1997.

**Figure 2:**
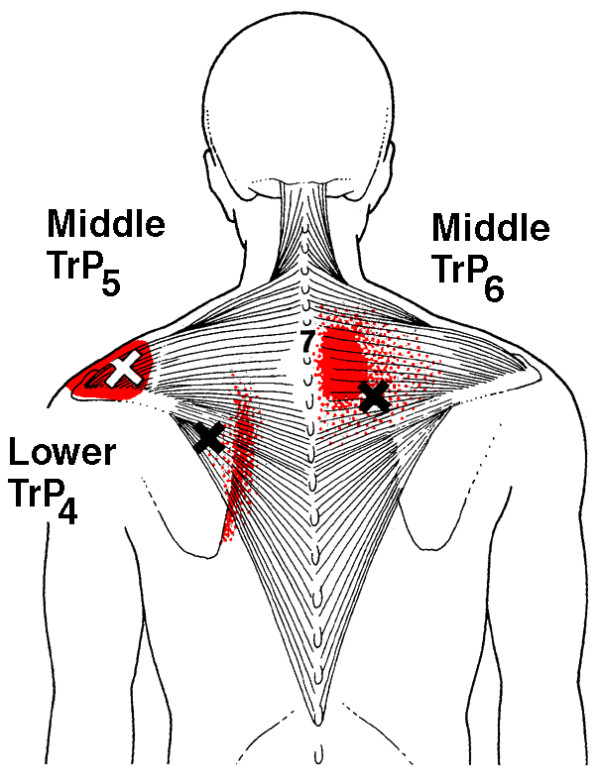
**Referred pain patterns (red) from the upper and middle trapezius muscle MTrPs (Xs), according to Simons et al**. Illustrations courtesy of LifeART/MEDICLIP, Manual Medicine 1, Version 1.0a, Lippincott, Williams & Wilkins, 1997.

**Figure 3:**
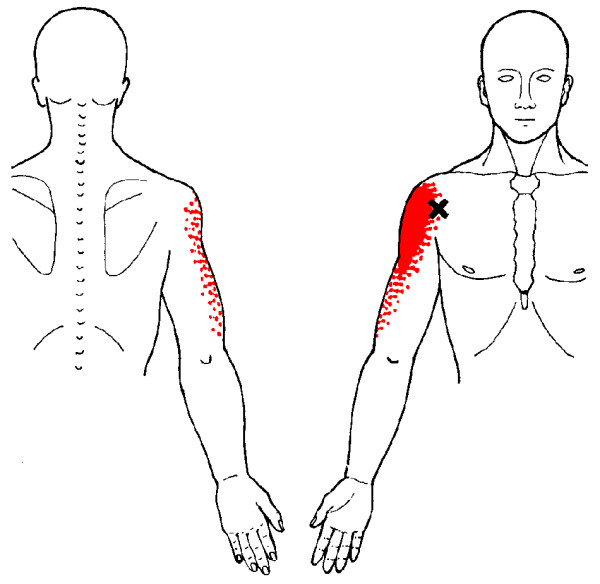
**Referred pain patterns (red) from the anterior deltoid muscle MTrPs (Xs), according to Simons et al**. Illustrations courtesy of LifeART/MEDICLIP, Manual Medicine 1, Version 1.0a, Lippincott, Williams & Wilkins, 1997.

**Figure 4:**
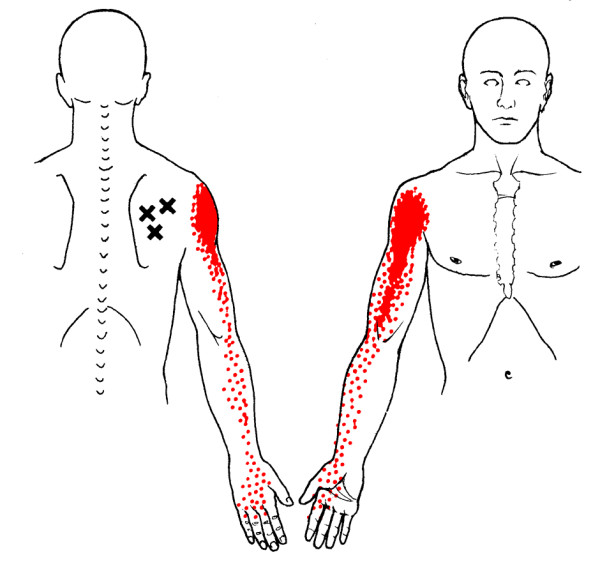
**Referred pain patterns (red) from the infraspinatus muscle MTrPs (Xs), according to Simons et al**. Illustrations courtesy of LifeART/MEDICLIP, Manual Medicine 1, Version 1.0a, Lippincott, Williams & Wilkins, 1997.

In clinical practice, identification of MTrPs is usually performed by palpation. In a recent study [35], we confirmed that this technique is a reliable method for detecting MTrPs in shoulder muscles. Although prevalence studies are sparse [36-42], based on clinical experience, MTrPs seem to be associated with shoulder pain, disability, and dysfunction [43-45]. Still, little is known about the impact of MTrPs on pain and functioning in patients with shoulder disorders [46]. Because MTrPs refer pain to the shoulder, they may contribute substantially to the clinical picture of shoulder pain (Figure 1, 2, 3 and 4). Experimental muscle pain, clinical muscle pain, and MTrPs have all been shown to alter motor activation patterns in a similar manner as the kinematic disturbances seen in shoulder pain patients often referred to as SIS [47-49].

The aim of this study was to determine the prevalence of MTrPs and the correlation between MTrPs and pain and functioning, in a sample of patients presenting with chronic, non-traumatic unilateral shoulder complaints.

## Methods

### Study design

This observational study was embedded in a clinical trial (registered at current controlled trials ISRCTN75722066) addressing a specific treatment of patients with shoulder pain [50]. The Committee of Human Research of the region Nijmegen-Arnhem, the Netherlands, has approved the study protocol [CMO 2007/22].

### Study Participants

Study participants were recruited from patients participating in a controlled trial investigating the effectiveness of physical therapy on patients with unilateral, non-traumatic shoulder pain. This study was conducted at a primary care practice for physical therapy, which specializes in the treatment of patients with disorders of the shoulder, the neck, and upper extremities. A power analysis was performed prior to beginning this study, and it was calculated that 104 subjects were needed for the clinical trial.

All patients who contacted the practice for non-specific shoulder complaints from September 2007 until September 2009 were requested to participate in the study. The inclusion criteria were 1) age between 18 and 66 years; 2) unilateral non-traumatic shoulder pain; and 3) duration of symptoms of more than six months. Patients were excluded from the study if they presented with a prior diagnosis of shoulder instability, shoulder fractures, any systemic diseases, or a medical history or examination suggestive for the presence of neurological disease, internal diseases, or psychiatric disorders. All patients signed a written informed consent before participating in the study.

### General Applicability

To determine the potential general applicability of this study to primary care shoulder pain patients, we searched for Dutch studies conducted on primary care patients from 1995 until 2009. Eight studies were found and sociodemographic data (age, gender, education level, and duration of shoulder pain) were analyzed and compared to the current study population [2,5,51-55].

### Measures

At baseline, age, gender, hand dominance, and education level were recorded. For comparison reasons we classified the education level as high education (university and higher vocational school), medium education (middle vocational school and higher or middle general secondary school), and low education (lower vocational school, lower general secondary school, primary school, or no education) [54]. Shoulder-pain related data (duration of shoulder-pain, recurrence rate and location of the complaints) were collected and the study subjects were asked to complete a set of standardized self-report measures, including the Disabilities of the Arm, Shoulder, and Hand outcome measure - Dutch Language Version (DASH-DLV), Visual Analogue Scale for Pain (VAS-P) and the Beck Depression Inventory- Second Version- Dutch Language Version (BDI-II-DLV) [50]. The BDI-II-DLV is used to discriminate between patients with major depression and those with only minor depressive feelings or no depression, which may be a confounding factor. The BDI-II has good predictive value, is widely accepted, and is commonly used in both clinical and experimental research. A BDI-II-DLV score equally or ≥ 21 indicates major depression (specificity 78.4%) [56].

For every study participant, one of the two available observers measured the passive range of motion (PROM) of the shoulder in flexion, internal and external rotation, abduction, and (horizontal or cross-body) adduction with a handheld digital inclinometer (The Saunders Group Inc, Chaska, MN). Range of motion was expressed in degrees and presented as the sum of the value measured for the non-affected shoulder minus the value measured for the affected shoulder. A positive value means that the affected shoulder had impaired range of motion as compared to the non-affected shoulder.

Next, the observer examined each subject for the presence of MTrPs in the shoulder muscles of their affected shoulder according to the guidelines outlined in Simons et al [26]; the non-affected shoulder was examined as a control. Following these guidelines, an MTrP is defined as: a nodule in a taut band that is extremely painful upon compression, and may produce referred pain or sensations. MTrPs were classified as either 'active' when the pain was recognized by the patient as a familiar pain, and 'latent' when the observer found a firm nodule in a taut band, which was painful on compression, but did not produce a recognizable pain. The inter-examiner reliability of trigger point palpation has been established in several studies [35,57,58]. All 17 muscles that are known to produce pain in the shoulder or may result in dysfunction of shoulder muscles were systematically examined and the number of muscles with MTrPs in the affected shoulder was counted, regardless of the number of MTrPs per muscle (Table 1). The two observers were physical therapists, each with 30 years of clinical experience in primary care practice. Both observers had attended an extensive, postgraduate course on MTrP diagnosis and therapy and had more than 5 years experience in identifying MTrPs and treating patients with MTrPs prior to the start of the study.

**Table 1:** List of muscles examined for presence of MTrPs


upper trapezius muscle	middle trapezius muscle	lower trapezius muscle
Infraspinatus muscle	supraspinatus muscle	subscapularis muscle
teres minor muscle	teres major muscle	anterior deltoid muscle
middle deltoid muscle	posterior deltoid muscle	pectoralis major muscle
pectoralis minor muscle	biceps brachii muscle	triceps brachii muscle
scalene muscles	subclavius muscle	

The DASH-DLV is a widely used multidimensional (physical, emotional and social) 30-item self-reporting questionnaire that focuses on physical function, pain and other symptoms. DASH-DLV scores ranges from 0 to 100, with higher scores indicating greater disability. DASH is a reliable and valid questionnaire, with good to excellent intra- and inter-rater reliability, and good correlation with the Shoulder Pain and Disability Index. Because of these advantages, DASH is considered to be one of the best questionnaires available for shoulder symptoms (http://www.dash.iwh.on.ca/) [59,60].

The VAS-P is a self-report scale consisting of a 100 mm horizontal line anchored by word descriptions on each side [61]. VAS-P can be used to measure pain current pain levels (VAS-P1), the average pain over the last 7 days (VAS-P2), and the most severe pain over the last 7 (VAS-P3)). VAS-P scores ranges from 0 (no pain) to 100 (the worst pain imaginable). The Visual Analogue Scale has properties consistent with a linear scale for patients with mild to moderate pain.

Data was collected and transferred to a worksheet by a research assistant (who was not involved in the physical examination or palpation of MTrP).

### Data analysis

Frequency distributions, means, medians, standard deviations, and 95% confidence intervals were calculated for descriptive purposes. The Shapiro-Wilk *W *test was used to test for normality of the data. Because the number of muscles with MTrPs (active, latent and total) was not normally distributed we used the Spearman's rank-order correlation (ρ) test for all variables. For interpretation of the ρ-values, we used the classification proposed by Feinstein [62]. A correlation coefficient < 0.30 was considered to be indicative of a poor correlation. A correlation coefficient ≥ 0.30 and ≤ 0.70 was considered to be indicative of moderate correlation, and a correlation coefficient ≥ 0.70 was defined as substantial or a good correlation. To test for differences in age, gender distribution, and education level between the current study population and study populations from Dutch shoulder studies (from 1995 until 2009), we used a one sample T-test. The α level for statistical significance was set at 0.05. All analyses were performed using Systat 12 or Sigmastat 3.1 for Windows (Systat Software, Inc. Chicago, IL, USA).

## Results

**Figure 5:**
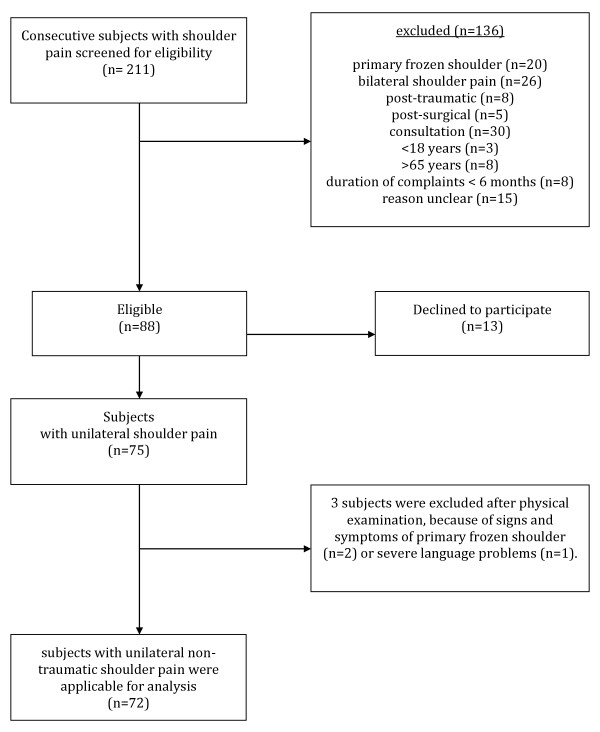
**Flow chart showing a schematic summary of patient participation in this study**.

A flowchart describing patient participation is depicted in Figure 5. Out of 211 patients who were treated for shoulder disorders, between September 2007 and September 2009, 72 patients (50 females and 22 males; mean age 43.9 years, SD 12.3; 95% CI 41.0 to 46.0) presented with unilateral, non-traumatic shoulder complaints, met the study inclusion criteria, and agreed to participate in this study. Twenty-six subjects were suffering from their first episode of shoulder pain, while for 19 subjects this was their second episode. The remaining 27 subjects had suffered from ≥ 3 episodes of shoulder pain. Study participants' characteristics are summarized in Table 2. A comparison of data obtained from the present study with data from previous Dutch studies is presented in Table 3. The mean age of the present study population was lower (*p  *< 0.05) and the proportion of female subjects was higher (*p  *< 0.05) compared to these other studies. In addition, the current study population was more highly educated (*p  *< 0.05) than the previous study populations for which educational data was reported [3,5,52]. Comparison of the duration of shoulder pain was not possible because different classifications were used.

**Table 2:** Characteristics of patients participating in this study (n = 72)

Characteristics	*n (%)*	*mean (SD; 95% CI); median*
Age (years)		43.9 (12.3; 41.0 - 46.8); 45.0
Gender, female	50 (69.4)	
Duration of shoulder pain		
6-9 months	17 (23.6)	
9-12 months	14 (19.4)	
1-2 years	13 (18.0)	
2-5 years	14 (19.4)	
>5 years	14 (19.4)	
Recurrence rate		
1^st ^episode	26 (36.1)	
2^nd ^episode	19 (26.4)	
3^rd ^> episode	27 (37.5)	
Hand dominance, left-handed	4 (5.6)	
Side of complaints right	48 (66.7)	
DASH-DLV (0 - 100)^a^		30.8 (14.1; 27.5 - 34.1); 28.3
VAS-P1 (0-100)^b^		30.0 (23.9; 27.0 - 39.9); 30.0
VAS-P2 (0-100)^b^		42.1 (17.7; 37.4 - 50.0); 40.0
VAS-P3 (0-100)^b^		56.6 (19.8; 51.2 - 61.9); 57.0
BDI-II-DLV (0 - 63)^c^		6.1 (6.0; 4.7 - 7.6); 5.00
0-13	68 (94.4)	
14-19	3 (4.3)	
20-28	0 (0.0)	
28-63	1 (1.4)^d^	

^a ^Higher Dash-DLV (Disabilities of the Arm, Shoulder and Hand outcome measure- Dutch Language Version) scores mean more disability with a maximum of 100 (range from 0 to 100)[59].

^b ^Higher VAS-P scores (Visual Analogue Scales for Pain) mean more pain, with a maximum of 100 (range from 0 to 100). VAS-P1 represents the current pain score, VAS-P2 represents the average pain score over the past seven days, and VAS-P3 represents the most severe pain score over the past seven days.

^c ^Higher scores on the BDI-II-DLV (Beck Depression Inventory-second edition- Dutch Language Version) mean more symptoms of depression. Clinical interpretation of scores is accomplished through criterion-referenced procedures utilizing the following interpretive ranges: 0-13 minimal depression; 14-19 mild depression; 20-28 moderate depression; and 29-63 severe depression[79].

^d ^One patient scored 45 points, which is indicative of major depression. This high score was due to a major event that happened on the day of inclusion in the study.

**Table 3:** Socio-demographic characteristics of the current study population and eight other Dutch shoulder research study populations

	Current study N = 72	Van der Windt 1996 N = 335	De Winter 1999 N = 201	Winters 1999 N = 101	Bot 2005 N = 281	Bergman 2005 N = 71	Kuijpers 2006 N = 492	Feleus 2008 N = 682	Reilingh 2008 N = 587
**Age (years,± SD)**									
	43 (12.3)	49.6 (14.4)	48 (12)	47.3 (15.4)	49.2 (13.8)	47.8 (11.8)	52 (14)	45*	49.5 (14.7)† 51.9 (13.9)‡ 52.9 (13.3)¶
**Gender (%)**									
female	69	56	66	58	63	52	50	52	50
**Education level**									
Low	6	NA	NA	NA	44	NA	NA	36	36
Medium	47	NA	NA	NA	42	NA	NA	36	41
High	47	NA	NA	NA	14	NA	NA	28	23
**Duration of shoulder pain (month)**									
< 3 m	0	85	26	75	66	70	60	74	59
3-6 m			16			30	40		41
> 6 m	100	15	55	25	34		26		

*Feleus reported the median instead of the mean age

†Mean age (±SD) of the acute pain group (< 6 weeks)

‡Mean age (±SD) of the subacute pain group (6-12 weeks)

¶Mean age (±SD) of the chronic pain group (> 3 months)

NA (not available). It was not possible to derive these data from the papers.

### Prevalence of muscles with myofascial trigger points per subject

**Figure 6:**
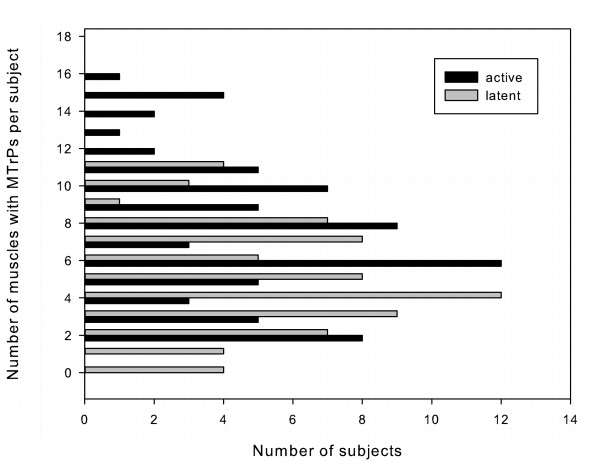
**The number of active (black bar) and latent (grey bar) of MTrPs per subject**. The X-axis shows the number of subjects, and the Y-axis shows the number of MTrPs per subject (n = 72).

Muscles containing active MTrPs were found in all 72 subjects. The median number of muscles with active MTrPs per subject was 6 (range 2 to 16). Muscles containing latent MTrPs were found in 67 subjects. The median number of muscles with latent MTrPs per subject was 4 (range 0 to 11). Figure 6 shows the frequency distribution of active and latent MTrPs per subject. Neither active MTrPs nor latent MTrPs were normally distributed (Shapiro *W *= 0.95; *p *< 0.05; *W *= 0.96; *p <*0.05 respectively).

### Prevalence of myofascial trigger points by muscle

Active MTrPs were found in the infraspinatus muscle in 56 subjects and in the upper trapezius muscle in 42 subjects. In addition, active MTrPs were highly prevalent in the middle trapezius (n = 31), anterior deltoid (n = 34), middle deltoid (n = 36), posterior deltoid (32), and teres minor (n = 34) muscles.

**Figure 7:**
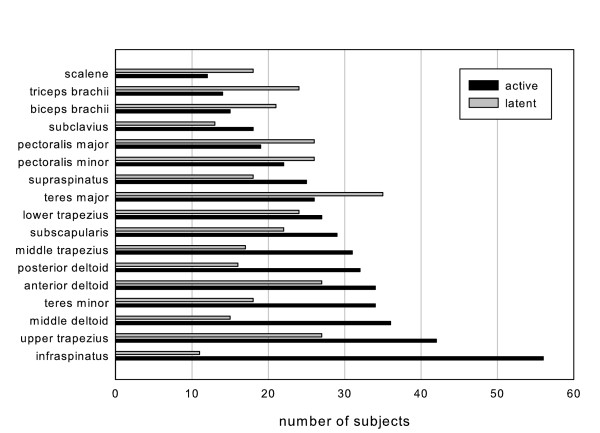
**The number of subjects with active (black bar) or latent MTrPs (gray bar) per muscle**. The X-axis shows the muscles that were examined for identification of MTrPs, and the Y-axis shows the number of subjects with MTrPs (n = 72).

Latent MTrPs were found in the infraspinatus muscle in 11 subjects and in the upper trapezius in 27 subjects. Latent MTrPs were found in the teres major muscle in 35 subjects and in the anterior deltoid muscle in 27 subjects. Figure 7 presents the distribution of active and latent MTrPs per muscle.

### DASH-DLV, VAS-P, BDI-II-DLV, and PROM

The mean score on the DASH was 30.8 (SD 14.1; 95% CI 27.5 to 34.1). Mean VAS-P scores were follows: the VAS-P score for 'current pain' (VAS-P1) was 30 (SD 23.9; 95% CI 27.0 to 39.9), for 'average pain in the last seven days' (VAS-P2) was 42.1 (SD 17.7; 95% CI 37.4 to 50.0) and for 'for the most severe pain in the last seven days' (VAS-P3) was 56.6 (SD 19.8; 95% CI 51.2 to 61.9). The mean PROM score, calculated as the sum the PROM value measured for the non-affected shoulder minus the PROM value measured for the affected shoulder, was 32.4 degrees (SD 34.8; 95% CI 24.2 to 40.6), where a positive value indicates that the affected shoulder has a impaired range of motion. Both DASH and PROM scores were normally distributed (*W *= 0.97; *p <*0.05 and *W = *0.91; p < 0.05 respectively). VAS-P1, VAS-P2, and VAS-P3 scores were also considered to be normally distributed, although the Shapiro-Wilk test did present borderline results for VAS-P2 and VAS-P3.

### Correlation between the number of muscles with MTrPs and pain and disability scores (DASH-DLV, VAS-P)

**Figure 8:**
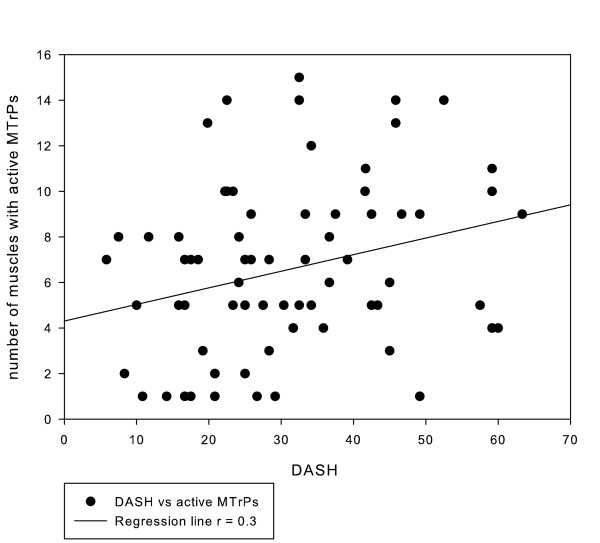
**Scatterplot of DASH scores versus the number of muscles with active MTrPs**. The regression line shows a weak positive correlation (r = 0.3), indicating that increasing numbers of active MTrPs have only a moderate effect on DASH scores.

The number of muscles with active MTrPs only moderately correlated with the DASH-DLV (ρ = 0.30; *p <*0.05) and VAS-P1 scores (ρ = 0.33;*p  *< 0.05), and poorly correlated with VAS-P2 (ρ = 0.28; *p <*0.05) and the duration of the shoulder pain (ρ = 0.26, *p <*0.05). We were unable to detect statistically significant correlations between the number of muscles with MTrPs (either active or latent) and VAS-P3 (ρ = 0.09; *p >*0.05) or the PROM (ρ = 0.13; *p >*0.05) scores. Table 4 provides an overview of the correlations and Figure 8 shows a scatterplot of DASH scores versus the number of active MTrPs.

**Table 4:** Correlation matrix of the current study population (n = 72)

	MTrPs	Active MTrPs	Latent MTrPs	DASHDLV	BDI-II DLV	VAS P1	VAS P2	VAS P3	Duration
MTrPs	-	0.65*	0.11	0.29*	0.22	0.44*	0.31*	0.06	0.26*
AMTrPs		-	-0.64*	0.30*	0.16	0.33*	0.28*	0.01	0.12
LMTrPs			-	-0.12	0.02	-0.02	-0.06	0.04	0.04
DASH-DLV				-	0.35*	0.66*	0.58*	0.27*	0.05
BDI-II-DLV					-	0.33*	0.18	0.07	0.13
VAS-P1						-	0.68*	0.35*	0.18
VAS-P2							-	0.57*	0.18
VAS-P3								-	-0.10
Duration									-

The data represent Spearman's rank correlation coefficient. Correlation coefficients between the number of muscles with myofascial trigger points (MTrPs), the number of muscles with active MTrPs (AMTrPs) and the number of muscles with latent MTrPs (LMTrPs), the DASH (Disability of the Arm, Shoulder and Hand) outcome measure- Dutch Language Version (DASH-DLV), the Beck Depression Inventory-second version- Dutch language Version (BDI-II-DLV), the Visual Analogue Scales for current pain (VAS-P1), the average pain over the last seven days (VAS-P2), the most severe pain over the last seven days (VAS-P3) and the duration of shoulder pain (Duration), are given (* p < 0.05).

## Discussion

### Prevalence of MTrPs

All subjects with unilateral, chronic, non-traumatic shoulder pain presented with multiple shoulder muscle MTrPs. In addition, MTrPs were found in all 17 muscles examined. However, the number of shoulder muscles with MTrPs appeared to vary greatly among subjects. In particular, MTrPs were most frequently located in the infraspinatus and upper trapezius muscles, in agreement with results from Skootsky [37] and Simons [26], who found that infraspinatus muscles were frequently associated with myofascial shoulder pain shoulder. There are very few other prevalence studies in the literature, and to the best of our knowledge, this is the first extensive report on the prevalence of MTrPs in patients with chronic, non-traumatic unilateral shoulder pain.

### Mean and median scores on DASH-DLV and VAS-P scores

The mean DASH-DLV score measured for the current study population is comparable with the mean baseline scores measured for other study populations for subjects with shoulder and arm pain [63-65]. According to Beaton[66] subjects (n = 200) with DASH scores < 23.6 are still able to perform all desired daily activities, although they may experience some discomfort. For comparison, in a study population from the US (n = 1706), the mean DASH score was 10.10 (SD 14.68) and in young active and healthy adults the mean DASH score was 1.85 (SD 5.99) [67]. Importantly, the DASH-DLV score primarily reflects the level of dysfunction with less emphasis on pain and other symptoms. While 23 items refer to the ability of the subject to perform activities, only 7 items assesses the severity of symptoms. Subjects with long-standing shoulder complaints may alter the way in which they perform activities by using compensatory movements. In addition, DASH-DLV does not discriminate between activities performed using the affected or non-affected arm, which may influence the magnitude of the disability and therefore the final DASH-DLV score. In support of this, several subjects in our study commented that their DASH score would have been different if the activities in question were related to the affected arm.

### Correlation between number of muscles with MTrPs, DASH-DLV scores, and VAS-P scores

The number of muscles containing active MTrPs moderately and positively correlated with DASH-DLV, VAS-P1, VAS-P2 scores, and the duration of the shoulder pain, suggesting that the number of muscles with active MTrPs explained only 10% of the variation of the outcome measures, including pain and disability. In addition, other clinically relevant factors may have contributed to the primary and secondary outcome scores. First, although we did not measure the pain intensity at the MTrP, this may have a significant impact on pain and functioning. Hidalgo et al found that patients with shoulder pain had a larger number of both active and latent MTrPs than healthy subjects. They also found that active MTrPs were associated with greater pain intensity, and that lower Pain Pressure Thresholds (PPT) were reported for active MTrPs compared to latent and patients with shoulder pain displayed lower PPT than healthy subjects [49]. Second, in this study we did not take into consideration the number of MTrPs per muscle, which may have contributed to the moderate correlation observed between the number of muscles with MTrPs and the DASH-DLV and VAS-P scores. The total number of muscles with MTrPs was poorly but positively correlated with the duration of the complaints, indicating that the number of shoulder muscles with MTrPs may increase over time regardless of whether the MTrPs were active or latent. Finally, because one of the characteristics of the DASH-DLV score is, that it does not discriminate between the affected and the non-affected shoulder, one could speculate that patients with chronic shoulder pain may develop strategies to overcome pain and disability caused by their shoulder disorder, for instance by using the non-affected arm, resulting in decreased DASH-DLV and VAS-P scores. All these factors may have a substantial influence on the correlation coefficient. Although the number of shoulder muscles with active MTrPs correlates only moderately with the various outcome measures, this does not imply that MTrPs are clinically unimportant. Future studies of chronic shoulder pain examining the total number of trigger points and their pressure sensitivity in the muscles studied could substantially impact the magnitude of the effect of presence of myofascial trigger points on shoulder pain and disability.

### Clinical implications

To date, unilateral shoulder pain has mainly been proposed to be due to either the presence of inflammation in the subacromial tendons and bursae, or degenerative rotator cuff ruptures (diagnosed using modern imaging techniques, such as MRI or sonography). Although these pathological structures may cause pain, it is also known that similar abnormalities have been found in asymptomatic shoulders.

Active MTrPs, which are painful spots that produce familiar shoulder pain during contraction, stretching or compressing, these MTrPs may provide an alternative explanation for shoulder pain, which is independent of the presence of subacromial abnormalities. According to Simons, Travell and Simons [26], MTrPs within the infraspinatus muscle (which were most prevalent) cause pain in the anterior and middle deltoid regions which expands into the frontal upper arm, as well as referred pain and referred sensations felt in the wrist and the hand. In addition, internal rotation and cross-body adduction may be impaired, which is often the case in patients with shoulder pain. Both experimentally induced and spontaneous muscle pain lead to an aberrant motor activation pattern that is also present in patients with shoulder pain [68,69]. Although latent MTrPs are not usually an immediate source of pain, they can elicit referred pain when mechanically stimulated, or during sustained or repeated muscle contraction. In addition, latent MTrPs may disturb normal motor recruitment patterns and movement efficiency. Lucas et al. showed that subjects who received myofascial dry needling, followed by passive muscle stretching to remove latent MTrPs, showed normalized motor activation patterns within 20 to 30 minutes following the treatment [48]. Therefore, it is reasonable to expect that treatment of MTrPs may lead to normalization of motor activation patterns and may facilitate spontaneous recovery of shoulder pain, either without exercising or by making exercise more effective.

Based on the results of this study, we propose that an alternative approach may be indicated for the assessment and management of patients with chronic, non-traumatic shoulder pain. Current treatment regimens consist primarily of pharmacological interventions, including anti-inflammatory medications, or muscle strengthening exercises. If MTrPs are one of the main reasons for shoulder pain (active MTrPs) and altered motor activation patterns (active and latent MTrPs), as several authors have proposed, then anti-inflammatory treatment [26,48,70] and muscle strengthening exercises should not be the treatment of first choice. Instead, the treatment should begin with MTrP inactivation. Manual techniques, including manual compression of the MTrP, known as ischemic compression or trigger point release, trigger point dry needling or injection therapy are used to inactivate MTrPs. After MTrP inactivation, muscle stretching and relaxation exercises, heat applications, dynamic exercises to improve range of motion and muscle reconditioning are instructed as appropriate. This therapy is accompanied with a gradual increase in daily activities.

If the above hypothesis is true, treatment of MTrPs could provide an innovative, promising therapy for shoulder pain. This study shows the results of patients' characteristics for a sample of patients with chronic, unilateral non-traumatic shoulder pain, who were recruited for a randomized clinical trial to study the results of MTrPs directed interventions by physical therapists in this group. The results of this study are published[71].

### General Applicability

We compared sociodemographic data from the current study population with similar data from several other Dutch shoulder pain research studies. Because none of these studies investigated MTrPs, we made this comparison to see whether there was reason to expect that the high prevalence of MTrPs we observed was unique to our population. In our study population more females were included, and the subjects were significantly younger and more highly educated than subjects from the other Dutch populations, although a specific explanation for these differences is lacking. There is no reason to suspect that educational levels correlate with the number of MTrPs and awareness of educational levels is mainly important for effectiveness studies, because they may impact the patients' motivation and compliance [72,73]. However, increased age may also be associated with increased number of MTrPs [74]. Because the subjects of the present study were younger, and musculoskeletal complaints tend to increase with age [74], there is no reason to suspect that we overestimated the prevalence of MTrPs in our population. On the other hand, there were more females in our study population, and females may be more prone to musculoskeletal disorders in general [75]. Thus, for this reason there may be a chance that MTrPs were slightly more prevalent in our study population [76-78]. Despite the above-mentioned differences, we conclude that our subjects are comparable with other patients with chronic shoulder pain and the findings of this study can be generalized to other patients.

### Strength and limitations of the present study

One of the limitations of our study is that we only examined patients with unilateral chronic shoulder pain and dysfunction, whereas MTrPs are thought to be responsible for both acute and chronic pain. It is conceivable that patients who developed chronic shoulder pain may have more MTrPs, and persistent MTrPs in the acute phase than patients who recover easily. In future research projects assessment of MTrPs in patients with acute shoulder problems should also be included. The small sample size is another limitation of this study. Before starting this study a power analysis was performed and it was calculated that 104 subjects would be needed for the clinical trial. After two years (one year more than originally planned, 72 subjects were enrolled in the study. For practical reasons, the study was completed with this smaller sample size, which may have influenced some of the results of this study. We used two observers in this study with identical clinical experience and post-graduate training on myofascial trigger point therapy. In addition, both observers found a comparable mean number of active MTrPs. Because there was no statistically significant difference in mean DASH scores obtained by the two observers, we consider both groups to be comparable and the findings obtained by both observers to be similar.

## Conclusions

This study demonstrates that MTrPs are very prevalent in patients with chronic unilateral, non-traumatic shoulder pain. In addition, the number of MTrPs is only moderately correlated with DASH-DLV outcome measures and VAS-P pain measures, indicating that MTrPs contribute to the clinical picture of common shoulder pain problems. We recommend that the MTrP examination and treatment should be considered for patients with shoulder pain in both future clinical studies and clinical practice.

## Authors' contributions

All authors have read, edited and approved the final manuscript. CB is the lead investigator, and developed the design of the study, carried out data-acquisition, analysis, interpretations, and prepared the manuscript as primary author. MW and RO provided advice on the study and the manuscript, and supervised the study. JD and BS provided intellectual contributions to the manuscript.

## Competing interests

The authors declare that they have no competing interests.

## Pre-publication history

The pre-publication history for this paper can be accessed here:

http://www.biomedcentral.com/1471-2474/12/139/prepub
